# P-1558. Short vs. Long Durations of Antibiotic Therapy for Pyelonephritis in Kidney Transplant Recipients

**DOI:** 10.1093/ofid/ofae631.1725

**Published:** 2025-01-29

**Authors:** Jordan Crew, Michael Veve, Mary Grace Fitzmaurice, George J Alangaden, Rachel M Kenney

**Affiliations:** Loyola University Medical Center, Chicago, Illinois; Henry Ford Health, Detroit, Michigan; Henry Ford Hospital, Detroit, Michigan; Henry Ford Health, Detroit, Michigan; Henry Ford Hospital, Detroit, Michigan

## Abstract

**Background:**

Emerging data suggests shorter durations of antibiotic therapy are as effective and safe as longer durations in general populations. The objective of this study is to compare outcomes of short (7-9 days) vs long (10-15 days) durations of antibiotic therapy for acute graft pyelonephritis in kidney transplant recipients (KTRs).

**Figure:**
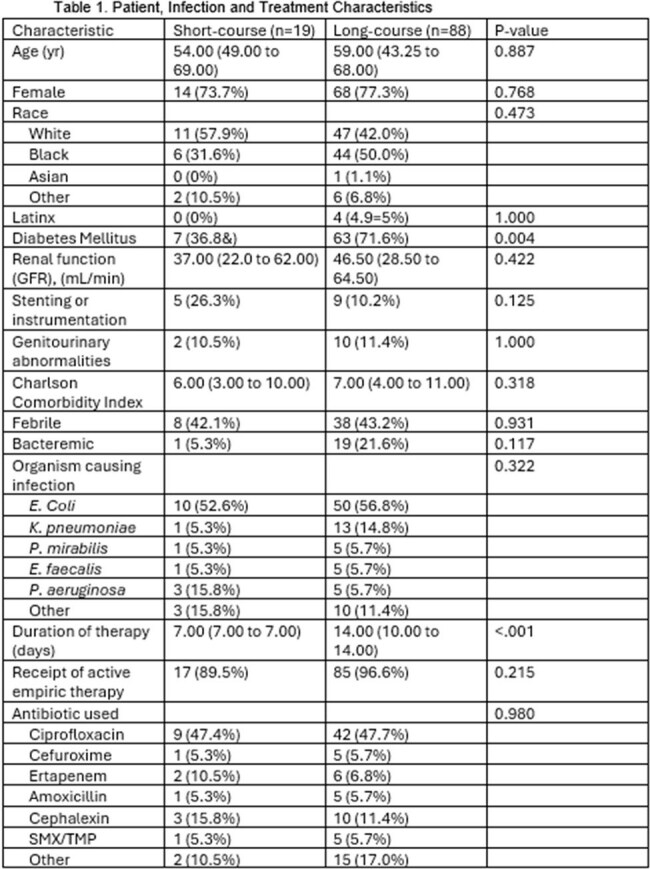

Summary of patient, infection and treatment characteristics.

**Methods:**

IRB-approved retrospective cohort comparing short (7-9 days) to long courses (10-15 days) of antibiotics. Inclusion: Adult patients, hospitalized from 1/1/14 - 12/31/23, history of kidney transplant, acute pyelonephritis, and receipt of at least 7 days of in vitro active antibiotic therapy. Exclusion: renal or perinephric abscess, prostatitis, ≤ 2 months post-transplant. Primary outcome: clinical success, defined as resolution of infection signs and symptoms and confirmed or presumed microbiologic success at end of therapy (EOT). Secondary outcomes: length of stay from pyelonephritis onset, continued resolution of signs and symptoms at 30 days, C. difficile infection within 30 days of EOT, acute rejection, and adverse effects.

**Figure:**
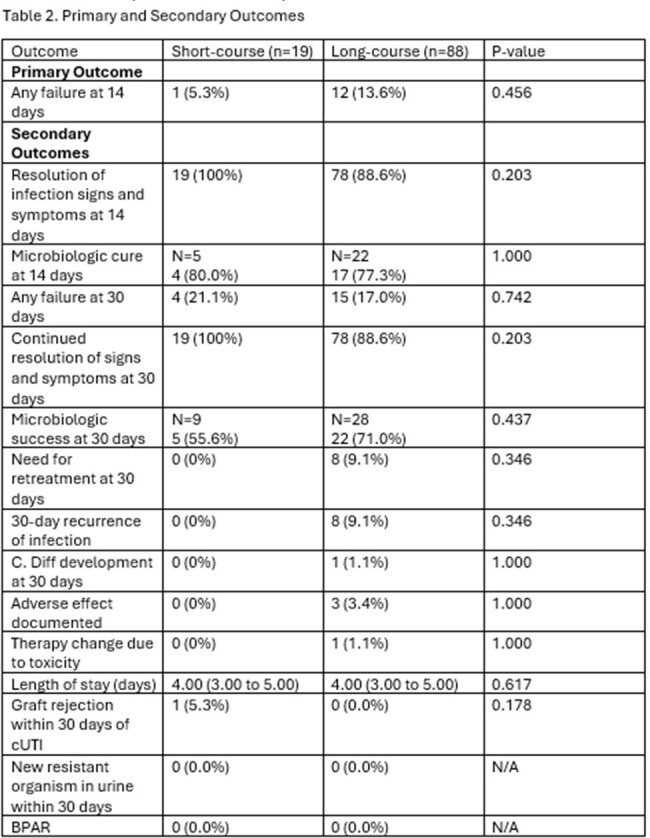

Summary of primary and secondary outcomes.

**Results:**

107 patients were included: 19 (17.8%) short-course and 88 (82.2%) long-course. Table 1 describes patient, infection, transplant, and treatment characteristics. There was 1 (5.3%) failure in the short-course group compared to8 (9.1%) failures in the long-course group at EOT (p=0.456). At 30 days, there were 4 (21.1%) failures in the short-course group and 15 (17.0%) in the long-course group (p=0.742). 30-day retreatment was required in 0 short-course and 8 (9.1%) long-course patients, p=0.346. No significant differences were detected for adverse effects: 0 vs. 3 (3.4%); C. diff: 0 vs. 1 (1.1%); or treated rejection: 1 (5.3%) vs. 0 in the short-course vs long-course groups, respectively.

**Conclusion:**

We did not detect differences in outcomes for pyelonephritis treatment in KTRs between those who received short compared to longer courses of antibiotic therapy. Further research is needed to solidify the place of short courses of antibiotic therapy in transplant recipients.

**Disclosures:**

**Rachel M. Kenney, PharmD, BCIDP**, Medtronic Inc: Spouse is an employee, stockholder

